# Chest Pain? An Unusual Presentation of Vertebral Osteomyelitis

**DOI:** 10.1155/2013/416498

**Published:** 2013-02-11

**Authors:** Cristian Landa, Stanley Giddings, Pramod Reddy

**Affiliations:** Department of Medicine, College of Medicine-Jacksonville, University of Florida, Jacksonville, FL 32209, USA

## Abstract

We present a case of vertebral osteomyelitis presenting as chest pain. The patient initially underwent a CT chest angiogram to rule out a pulmonary embolism, which incidentally showed a soft tissue vertebral mass at T3-T4 disk space. Subsequent thoracic vertebral MRI was consistent with osteomyelitis with cord compression. Tissue culture from a CT-guided biopsy grew MRSA. The patient was successfully treated with Vancomycin. This is a rare presentation of vertebral osteomyelitis which poses an interesting diagnostic challenge.

## 1. Case Presentation

**Figure 1:**
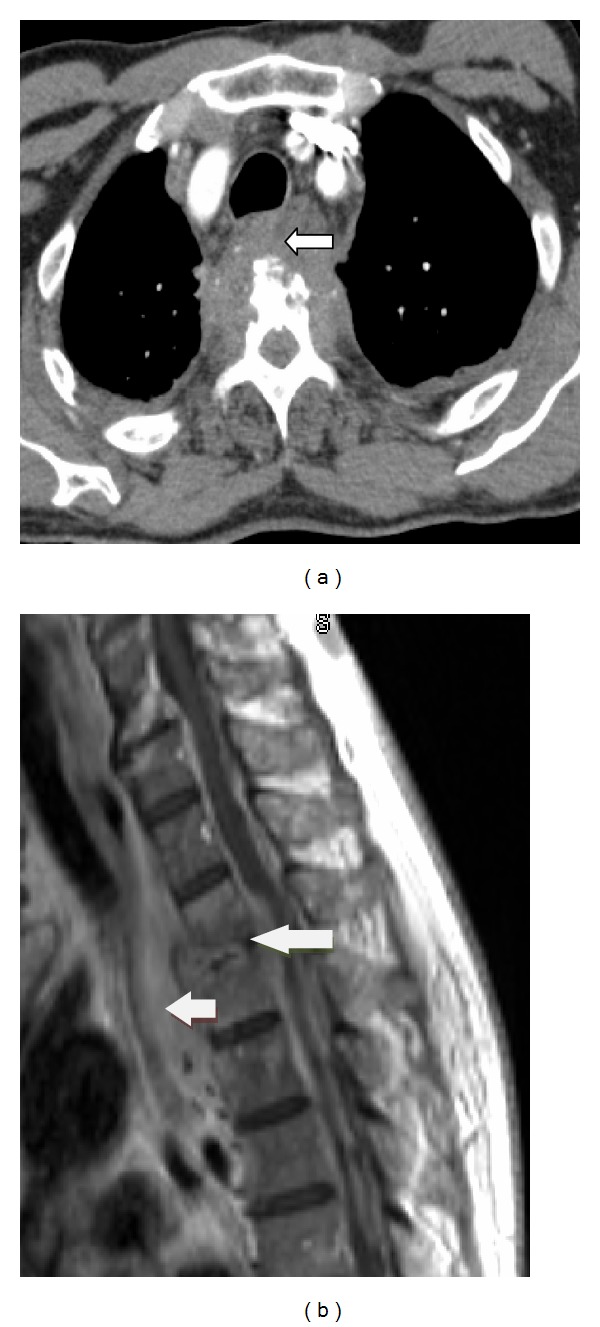
(a) CT angiogram chest showing a large soft tissue mass at the T3-T4 vertebral space with associated osseous destruction of the T4 vertebral body. (b) MRI of the thoracic spine, T1 weighted, showing T3-T4 disk space narrowing, endplate destruction, and marrow edema. There is also a soft tissue mass extending from the space anteriorly in the prevertebral space.

A 69-year-old African American male presented to the emergency department with a 4-day history of chest pain. He described the chest pain as band-like pressure across the chest, 10/10 in severity, constant, radiating to the back, worsened with coughing, and without relief by nitroglycerin. His past medical history included hypertension, chronic hepatitis C, prostate cancer s/p prostatectomy, a penile prosthesis removed the prior year due to infection, and several cystoscopies within the past year. Physical exam revealed a normotensive, afebrile patient with tenderness to palpation of the thoracic spine at T4 level. There were no focal neurologic deficits, and peripheral pulses were symmetric. Labs showed an elevated ESR without a leukocytosis. ECG showed normal sinus rhythm without any significant abnormalities. A CT angiogram of the chest ruled out a pulmonary embolism but showed a large soft tissue mass centered on the T3-T4 disk space. Subsequently, an MRI of the thoracic spine demonstrated osteomyelitis (Figure 1). Broad-spectrum IV antibiotics were started, and neurosurgery was consulted. A CT-guided biopsy showed mixed inflammation, and tissue culture grew MRSA. Antibiotics were deescalated to Vancomycin. ESR trended down with antibiotic therapy. Blood cultures remained negative. A transthoracic echocardiogram did not show any vegetation. The patient was discharged home on IV Vancomycin for a total of six weeks of therapy. 

## 2. Discussion

Vertebral osteomyelitis can pose a diagnostic challenge when unusual symptoms predominate on presentation. Its incidence has been estimated to be approximately 2.4 cases per 100,000 population [1]. It is mainly a disease of adults, predominantly those in their fifth decade of life with incidence increasing with increasing age [1, 2]. Predisposing factors include diabetes mellitus, malnutrition, substance abuse, HIV, malignancy, long-term steroid use, CKD, and cirrhosis [2]. It commonly results from hematogenous seeding, direct inoculation, or contiguous spread from an adjacent soft tissue infection [1, 2]. More than 50% of cases of vertebral osteomyelitis are caused by *Staphylococcus aureus* and *Streptococcus* species [3]. Back pain, fever in approximately 50% of cases, and muscular weakness are the most common presenting symptoms [4]. However, some uncommon signs and symptoms include chronic epigastric pain, lymphadenopathy, gait disturbances, and chest pain [5, 6]. Vertebral osteomyelitis has also been associated with illnesses such as subacute endocarditis, diffuse idiopathic skeletal hyperostosis, empyema, and pyomyositis, all of which can make its diagnosis more difficult and delayed [7–9]. A literature search provided four cases of vertebral osteomyelitis presenting with associated chest pain [10–13]. From these four case reports, only Sullivan et al. described a case presenting with pleuritic chest pain as the main complaint. We stipulate that the chest pain in our patient was caused by nerve root compression at T3-T4 level, which improved after treatment. To our knowledge, this is the only case where nonpleuritic chest pain was the main symptom without any accompanied muscular weakness or sensory deficits on exam. A rapid diagnosis was achieved due to the incidental finding of the vertebral mass on CT angiogram of the chest to rule out a pulmonary embolism. The MRI remains the preferred imaging modality. ESR and CRP are highly sensitive, and blood cultures are helpful in that if positive they preclude further invasive procedures [14, 15]. Antimicrobial treatment should be directed against an identified organism, which is possible in the majority of cases. The recommended duration of therapy ranges between 4 and 6 weeks [15]. Surgical intervention is usually not required for the exception of cases where an abscess needs to be drained and CT-guided catheter is not possible [16, 17]. Yoon et al. found that even in patients with culture negative vertebral osteomyelitis, good outcomes are usually achieved with 6 weeks of antimicrobial therapy and the values of CRP and ESR after four weeks of initiation of therapy provide useful information on the response to treatment [18]. 

The diagnosis of vertebral osteomyelitis is challenging, considering the rarity of disease, high prevalence of back pain in the general population, and variability of presenting symptoms. Diagnosis is based on clinical, laboratory, and radiographic data, and a high index of suspicion is required for its prompt recognition and improvement in disease outcome.
